# Conspicuous and cryptic reef fishes from a unique and economically important region in the northern Red Sea

**DOI:** 10.1371/journal.pone.0223365

**Published:** 2019-10-31

**Authors:** Calder J. Atta, Darren J. Coker, Tane H. Sinclair-Taylor, Joseph D. DiBattista, Alexander Kattan, Alison A. Monroe, Michael L. Berumen

**Affiliations:** 1 Red Sea Research Center, Division of Biological and Environmental Science and Engineering, King Abdullah University of Science and Technology, Thuwal, Saudi Arabia; 2 School of Aquatic and Fishery Sciences, University of Washington, Seattle, WA, United States of America; 3 Australian Museum Research Institute, Australian Museum, Sydney, NSW, Australia; 4 Trace and Environmental DNA (TrEnD) Laboratory, School of Molecular and Life Sciences, Curtin University, Perth, WA, Australia; Australian Bureau of Agricultural and Resource Economics and Sciences, AUSTRALIA

## Abstract

Al Wajh Bank in the northern Red Sea contains an extensive coral reef system that potentially supports a novel fish community. The large (1500km^2^) and shallow (< 40m depth) lagoon experiences greater temperature and salinity fluctuations, as well as higher turbidity, than most other Red Sea reefs. Since these conditions often influence coral community structure and introduce physiological challenges to its resident organisms, changes in reef-associated fishes are expected. We present critical baseline data on fish biodiversity and benthic composition for the Al Wajh Bank. Underwater visual census of conspicuous fishes and standardized collections of cryptobenthic fishes were combined to provide a comprehensive assessment of these fish communities. We documented 153 fish species and operational taxonomic units, including undescribed species, within 24 families on reefs largely dominated by hard coral and soft sediment (39% and 32% respectively). The families Pomacentridae and Gobiidae contributed the most towards fish diversity and abundance. Bray-Curtis dissimilarity distances among sampled sites suggest a distinctive fish community within the lagoon, and coefficients of variation for each species show high variation in their distribution across the lagoon. Species accumulation curves predict that additional sampling would document many more species throughout Al Wajh. Our findings provide the most extensive biodiversity survey of fishes from this region to date and record the condition of the reef prior to major coastal development planned to occur in the near future.

## Introduction

Coral reefs contain some of the most species-rich communities of marine fishes [1, 2], yet many poorly-studied reefs remain. For these reefs, assessing biodiversity provides a foundation for understanding and maintaining the health of local resources. The value of maintaining high biodiversity ranges from increasing the stability and resiliency of an ecosystem to providing greater access to biotic resources and building a reservoir of knowledge for future innovations [3–7]. However, globally coral reefs are facing rapid changes that will likely cause declines in biodiversity [8–12], therefore documentation of reef communities is critical for future management strategies, especially in poorly-studied regions such as the Red Sea.

Studies documenting reef fish communities in the Red Sea are limited when compared to the relatively accessible Great Barrier Reef and Caribbean reefs [13]. Coral reefs can be found along the entire coastline of the Red Sea between 16.5°N and 28.5°N, which experiences a variety of environmental conditions along a latitudinal gradient [14, 15]. Waters in the northern Red Sea remain relatively cool (up to 27°C at its most northern extremity in the Gulf of Aqaba) and nutrient-poor, whereas the southern Red Sea is warm (up to 33°C) and eutrophic due to an influx of nutrient-rich surface water from the Gulf of Aden during the summer months [16]. Nutrients such as silicates can also be supplemented by irregular aeolian input from Saharan and Arabian dust [17, 18]. With such high variability in environmental conditions, the Red Sea is expected to support high ecosystem diversity, which can in turn result in higher species diversity and ecological stability throughout the region [19]. While a pattern of heterogeneous fish communities is not well supported [16, 20], there remain reefs where these communities are undocumented. Al Wajh Bank in the northern Red Sea has not yet been surveyed but has the potential to support a novel community.

**Fig 1:**
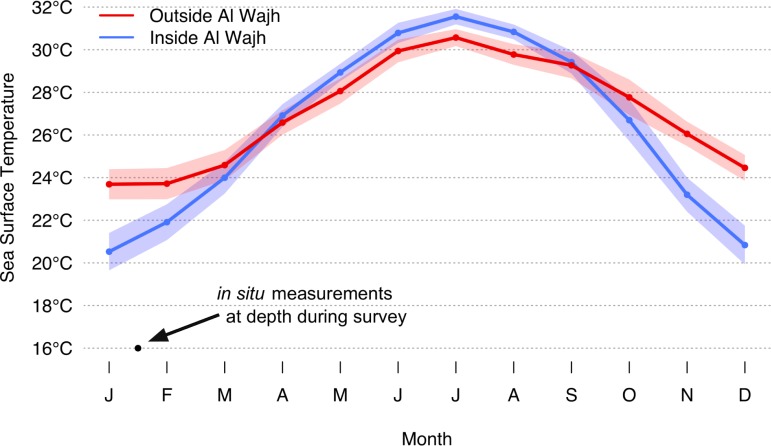
Moderate Resolution Imaging Spectroradiometer (MODIS) monthly sea surface temperature inside and outside Al Wajh Bank, averaged from June 2002 to May 2017. Transparent range indicates standard error. p < 0.05 (paired two-tailed t-test, α = 0.05) between inside and outside for all months except September.

Al Wajh Bank is a large and shallow barrier reef system situated in the northern to central Red Sea along the Saudi Arabian coastline between 25.28°N and 25.89°N (~75km linear distance and extending ~35km offshore) near the towns of Al Wajh and Umluj. The 1500km^2^ region contains roughly 50 islands scattered across a sandy basin with a maximum depth of ~30m. The extensive barrier reef nearly encloses the entire lagoon, with only three narrow channels deeper than 5m, between 100m to 900m wide, connecting to outside the lagoon. The reef’s bathymetry is unique among other Red Sea coastal systems, which are typically composed of isolated pinnacles extending vertically from the continental shelf up to 200m deep and therefore exposed to unrestricted water flow. There is little documentation on the hydrology within Al Wajh Bank, but its contained and shallow bathymetry suggests there is low wave action and water circulation. These properties interact with evaporation and aeolian dust resulting in an environment with high salinity and turbidity, and extreme annual temperature fluctuations. The annual change in sea surface temperature (SST) inside the lagoon is nearly double that from outside (Fig 1). Lagoon SST ranges from 30.5°C in the summer (1°C warmer than outside) to 20.5°C in the winter (3°C cooler than outside). Temperature profiles for the Red Sea show that even at depths surpassing 1000m, water temperature remains consistent at 21°C [21], however, *in situ* measurements from this study at < 9m depth were as low as 16°C inside the lagoon during winter. This extreme fluctuation can be explained by the shallow bathymetry of the Al Wajh Bank. Indeed, in the summer, the relatively small volume of water spread over a flat basin absorbs and stores thermal energy from solar radiation, while surface water is rapidly cooled by winds in the winter. Despite these extreme conditions, scleractinian corals form extensive reef structures in the region [22, 23], possibly due to relatively low human impact and adaptation to local environmental parameters [24]. In the Red Sea, these environmental conditions are only known from Al Wajh Bank, suggesting that the species composition of reef fishes could also be unique.

Plans to transform Al Wajh Bank into a major tourist destination are expected to commence in 2019 as a part of Saudi Arabia’s Vision 2030 [25, 26]. With the potential for reef fish communities to be impacted by development of the adjacent coastline, collecting baseline community data is vital for future monitoring of the region. In this study, visual surveys and ichthyocide surveys are combined to better capture taxonomic diversity by incorporating commonly detected fishes and cryptobenthic fishes. The latter are generally overlooked by traditional census methods because of their small size (< 50mm as adults) and cryptic nature [27]. Studies including both groups are lacking in this region. We therefore aim to (1) present the most comprehensive documentation of fish communities from Al Wajh Bank to date, and (2) examine both fish and benthic community data to explore its potential as a novel system within the Red Sea.

## Methods

### Survey sites and benthic properties

**Fig 2:**
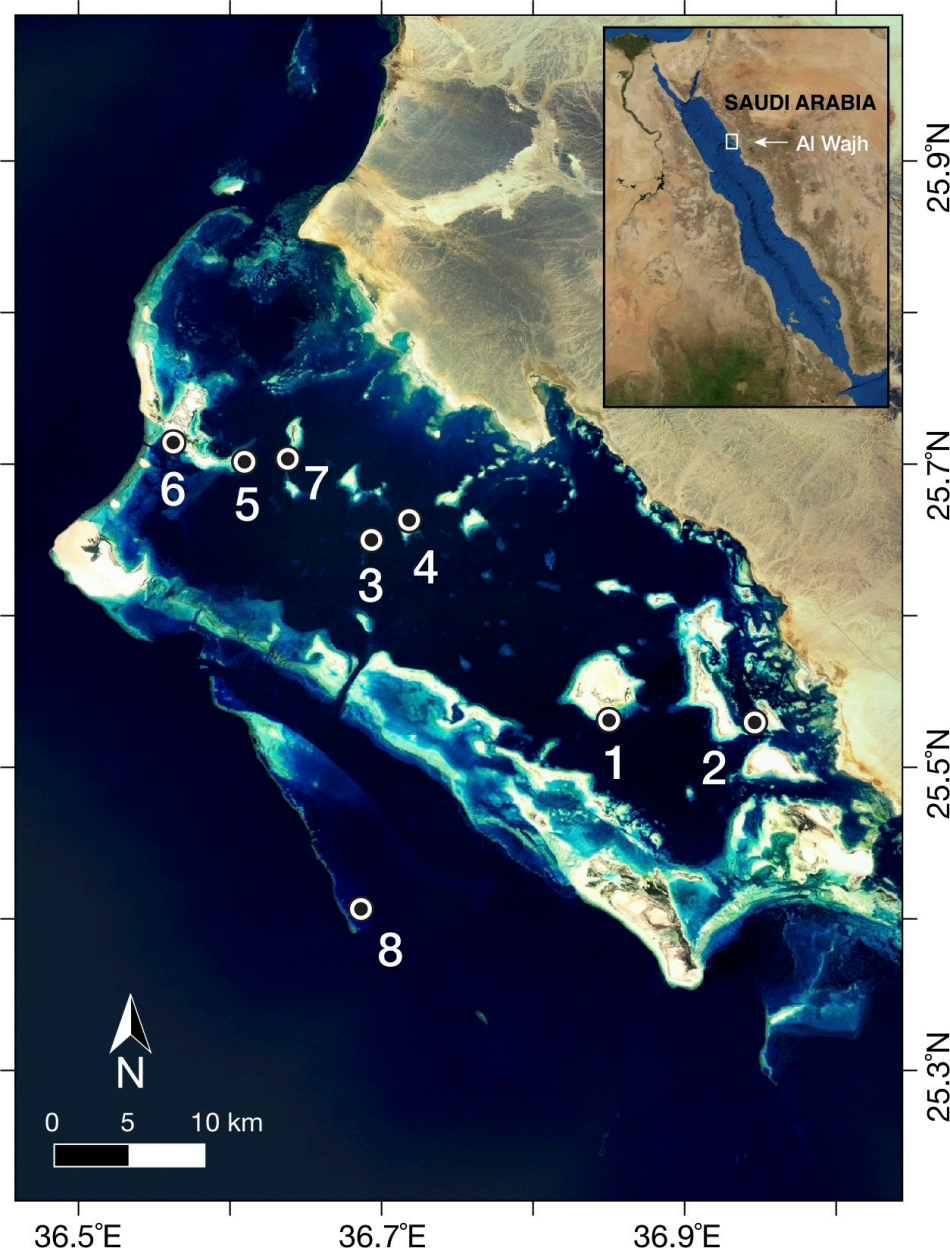
Map of Al Wajh Bank and sites surveyed. Created using ESRI World Imagery basemap. Sources: Esri, DigitalGlobe, GeoEye, i-cubed, USDA FSA, USGS, AEX, Getmapping, Aerogrid, IGN, IGP, swisstopo, and the GIS User Community.

Surveys were conducted over three days, from January 31st to February 2nd, 2016. Fish communities and benthic cover were surveyed at eight sites across the region (Fig 2) between 2.5m and 9m depth (Table 1) based on the maximum depth of the reef at each site. Data were collected from three replicated transects and one cryptobenthic collection station per site. Transects were 25m long, laid over continuous and patchy reef at a constant depth, and separated from one another by at least 5m. Cryptobenthic collection stations consisted of a 2 x 2 m^2^ PVC quadrate and were established near the start of the transects on the first relatively flat section of the reef matrix observed.

**Table 1:** Site location and benthic properties.

Site	Latitude	Longitude	Depth (m)	Height (cm)	Rugosity
1. River Island	25.53105°N	36.85006°E	5.7 ±0.2	67.0 ±6.08	0.55 ±0.05
2. Dolphin Spout Channel	25.52957°N	36.94616°E	5.7 ±1.0	43.6 ±3.38	0.65 ±0.02
3. The Black Hole	25.64999°N	36.69535°E	3.7 ±1.2	68.6 ±4.63	0.69 ±0.04
4. Amr's Drop Off	25.65683°N	36.71207°E	5.6 ±3.6	75.0 ±6.80	0.69 ±0.03
5. Umm Urumah	25.70152°N	36.60945°E	4.8 ±1.3	62.3 ±7.79	0.71 ±0.03
6. Umm Urumah (west)	25.71423°N	36.56246°E	2.5 ±1.5	44.0 ±3.05	0.65 ±0.07
7. Tane's Delight	25.70349°N	36.63802°E	3.6 ±0.1	71.3 ±2.90	0.71 ±0.01
8. Sea Moth Grotto	25.40652°N	36.68632°E	8.8 ±3.8	43.6 ±6.76	0.74 ±0.03

Rugosity = ratio of distance over reef contour to equivalent linear distance. Height = vertical distance occupied by reef structure. Depth and measured along UVS transects. Height and rugosity measured at collection stations. Standard error calculated for all values.

Benthic surveys were conducted along the transects. At fixed points every 0.5m (for a total of 50 points) along each transect, the type of benthic organism or substrate directly beneath the transect was recorded. Benthic types were classified into seven major categories: hard coral, soft coral, dead coral, sponges, turf algae, rubble & pavement, and sand. Hard corals were further identified to family or genus. Reef rugosity and height were observed at the collection stations as a proxy for habitat complexity. Rugosity was calculated as the linear distance between the ends of a 2m chain laid across the contour of the reef divided by the length of the chain, replicated three times. Height was measured as the vertical distance from the substrate to the three highest points of the reef structure within the quadrate.

### Fish surveys

Underwater visual surveys (UVS) are a commonly employed survey method in reef fish studies [28] as they can be conducted relatively quickly and with minimal interference. They are, however, limited in scope to conspicuous fishes and the observer’s identification abilities while underwater. We conducted UVSs, 25m long and 4m wide, to target conspicuous fishes on each transect outlined above. All fishes observed within the transect area were visually identified and recorded along with an estimate of the number of individuals per species. Surveyors had the support of a predetermined list of reef fishes known from the Red Sea.

Ichthyocide surveys and other standardized methods for targeting cryptobenthic fishes are more laborious than UVSs, but they have been shown to significantly increase observed species richness [29, 30]. At each collection station, we enclosed the PVC quadrate with a fine mesh net. A mixture of 1 kg of 4% rotenone, 500 mL of ethanol, and 500 mL of liquid detergent was used as an ichthyocide within the targeted area. This method is based on that used by Coker et al. [29] for comparability. Collected fish were identified to the lowest taxonomic level possible using a combination of visual and genetic methods; photographs and tissue samples were taken immediately after collection. All tissue and specimens were preserved in 96% ethanol for vouchering and genetic analysis.

### Genetic identification

Due to the inherent difficulties associated with identifying cryptobenthic reef fishes, sequence data were obtained from specimens collected during the rotenone survey and were used in conjunction with visual identification to assign species names where possible. Reliable genetic data could not be obtained from all specimens, so a number were simply identified visually. Total genomic DNA was extracted from fin tissue samples using the HotSHOT protocol [31] and then stored at -20°C. The quality and quantity of extracted DNA was assessed using a NanoDrop spectrophotometer. Poor-quality DNA was re-extracted using the DNeasy Blood & Tissue Kit (Qiagen, Valencia, CA).

The mitochondrial cytochrome c oxidase-I (COI) gene has been shown to effectively discriminate between fish species [32]. This study employs a 655 base pair fragment of COI, which was amplified using the following primers: FishF2 5’-TGT AAA ACG ACG GCC AGT CGA CTA ATC ATA AAG ATA TCG GCA C-3’ and FishR2 5’-CAG GAA ACA GCT ATG ACA CTT CAG GGT GAC CGA AGA ATC AGA A-3’. For each sample, polymerase chain reaction (PCR) was performed on a 12.5μL mixture of 1μL extracted DNA, 6.25μL Qiagen MasterMix, 4.25μL H_2_O, and 0.5μL of each primer (10 μM), using the following cycling parameters: 95°C for 15min; 35 cycles at 94°C for 60s, 50°C for 60s, 72°C for 45s; 72°C for 10min; store at 4°C. PCR products were visually verified using a 1% agarose gel with SYBR® Green dye, then cleaned using Qiagen ExoSAP (1.2μL ExoSAP to 7μL PCR product; incubate at 37°C for 60min, 85°C for 15min), and then sequenced using the Sanger method [33] at the KAUST Bioscience Core Lab facility. Sequences were aligned, trimmed, and quality-checked in Geneious 6.1.7. Each sequence was queried against the National Center for Biotechnology Information online database and added to a developing genetic library of Red Sea fishes initiated by Isari et al. [34], which includes results from Coker et al. [29].

### Community analysis

Abundance data for UVS and ichthyocide surveys were scaled to fish-per-square-meter, then merged for all sites. Fishes described to the genus level were discarded from analysis if they could not be distinguished from another species within the genus to avoid double-counting species. For example, *Caesio sp*. was detected via UVS and *Caesio caerulaurea* was detected in the ichthyocide survey. However, the former was removed from analysis, as it has potential to be *C*. *caerulaurea*. To analyze community structure and variation across Al Wajh, we generated a non-metric multidimensional scaling (nMDS) plot with Bray-Curtis dissimilarity distances between sites [35, 36] and report the stress value, which inversely correlates with how well the multivariate data are represented on the two plot axes. Abundance data were square-root transformed to reduce the effect of highly abundant species.

We compared the coefficient of variation (CV) for the species contributing 80% of the total abundance to better understand species-level distributions. The CV was calculated by dividing the standard deviation by mean abundance, which allowed us to compare how localized (larger CV) or wide-spread (smaller CV) a species is. A species found at a single site will have a CV of 2.83 (the maximum value).

SIMPER analyses were used to compare fish communities from UVS and ichthyocide surveys to data collected from published surveys along the Saudi Arabian coastline of the Red Sea [20, 29]. Roberts et al. [20] combined their sites into groups of four, and each site contained three 50m long UVS transects of varying widths, 4m, 2m, and 1m, each targeting fishes of a different size-range. The 1m transects, which targeted fishes from the families Blennidae, Gobiidae, and Pseudochromidae, were removed from our comparison of conspicuous fishes as these families were not detected by our UVS method and nearly all members of the families exhibit cryptobenthic lifestyles [27]. Additionally, since the Roberts et al. [20] UVSs had a much larger sampling effort, we only report comparisons in which higher percent occurrence was seen in Al Wajh Bank. Abundance data were reduced to presence-absence in both cases to reduce sampling biases.

Sampling was assessed using rarefaction curves [37]. Curves scaled by site and by individuals observed were constructed for UVS transects, ichthyocide surveys, and the combined results using the R package vegan. True species richness throughout Al Wajh was estimated for both survey methods and the combined results using the jackknife estimator developed by Heltshe and Forrester [38]. Although other estimators exist, the above method is appropriate for species richness measured at different points within the region of interest.

### Ethics statement

This research was undertaken in accordance with the policies and procedures of the King Abdullah University of Science and Technology (KAUST). Permits for sampling in Saudi Arabian waters using the ichthyocide rotenone were obtained from the Saudi Arabian coastguard. No specific permissions were required, as the study did not involve endangered or protected species. We were unable to obtain ethics approval or a waiver because no ethics board or committee for working with animals existed within KAUST or the Kingdom of Saudi Arabia at the time of collection.

## Results

### Fish biodiversity and composition

This study documented 6427 individual fishes consisting of 153 distinct species and operational taxonomic units (OTUs), within 24 families (Table 2). OTUs were established for morphotypes that could not be assigned to a species. Fish survey data and a list of genetically identical OTUs between this study, Coker et al. [29] and Isari et al. [34] can be found in the supplementary material. UVS sampling yielded far more individuals than the ichthyocide sampling (a 570% difference), but only resulted in nine more species (an increase of 13%) despite sampling over an area 75 times larger. Additionally, the diversity and evenness indices were higher for ichthyocide samples, at 30% and 33%, respectively. The two survey methods also detected a significantly different assemblage of taxa, with only 20 species shared between the methods.

**Table 2:** Biodiversity metrics at each site, for the underwater visual survey (UVS), ichthyocide survey, and the combined dataset from both methods.

Site	Reef	Families	Genera	Species	Density	H’	1-λ	E_H_
UVS								
1	River Island	9	18	24	1.04	2.2	0.83	0.49
2	Dolphin Spout Channel	7	13	16	0.3	2.32	0.87	0.51
3	The Black Hole	9	18	24	1.46	1.78	0.68	0.39
4	Amr's Drop Off	11	23	27	1.28	2.23	0.83	0.49
5	Umm Urumah	13	27	37	3.42	1.6	0.7	0.35
6	Umm Urumah (west)	10	21	26	0.68	2.58	0.88	0.57
7	Tane's Delight	11	23	30	7.44	1.04	0.47	0.23
8	Sea Moth Grotto	17	41	55	3.58	1.98	0.68	0.44
** **	Al Wajh Total	24	57	92	2.4	2.59	0.86	0.57
Ichthyocide Survey								
1	River Island	5	12	14	14	2.17	0.83	0.49
2	Dolphin Spout Channel	4	8	8	4.75	1.91	0.83	0.43
3	The Black Hole	9	21	25	63.5	2.05	0.76	0.47
4	Amr's Drop Off	9	17	19	20.25	2.36	0.85	0.54
5	Umm Urumah	5	12	17	11.75	2.3	0.85	0.52
6	Umm Urumah (west)	9	25	31	33.5	2.68	0.89	0.61
7	Tane's Delight	10	21	23	43.25	1.93	0.73	0.44
8	Sea Moth Grotto	10	20	24	24.25	2.7	0.91	0.61
** **	Al Wajh Total	20	53	81	26.91	3.36	0.94	0.76
Combined Survey Methods								
1	River Island	12	24	33	14.29	2.31	0.84	0.46
2	Dolphin Spout Channel	10	19	22	5.05	2.08	0.84	0.41
3	The Black Hole	15	34	46	64.21	2.11	0.77	0.42
4	Amr's Drop Off	18	37	44	20.29	2.49	0.85	0.49
5	Umm Urumah	16	37	53	14.67	2.62	0.89	0.52
6	Umm Urumah (west)	17	44	55	34.19	2.76	0.89	0.55
7	Tane's Delight	17	37	48	50.19	2.17	0.78	0.43
8	Sea Moth Grotto	21	54	72	26.9	2.9	0.92	0.58
** **	Al Wajh Total	36	92	153	28.72	3.5	0.95	0.7

Density is in individual fishes per m^2^. H’ = Shannon diversity index. 1-λ = Simpson diversity index. E_H_ = Shannon’s equitability index.

**Fig 3:**
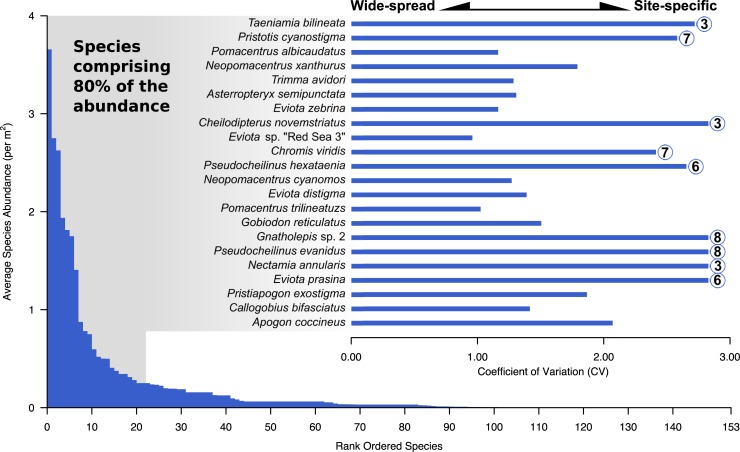
Rank ordered species abundance per transect. CV is shown for species comprising 80% of the total abundance in Al Wajh Bank. Survey site most associated with nine highly localized species are shown.

After combining data from both survey methods, the composition of fishes in Al Wajh Bank was highly skewed towards 22 species (14% of the species observed) comprising 80% of the total abundance (Fig 3), of which the majority were cryptobenthic species. Only seven of the listed species were observed in UVS transects. Note that on average, no species comprised more than four individuals per square meter, but there was high variation in how species were distributed between sites. The highest density species was *Taeniamia bilineata* (Apogonidae), but 97% of the individuals were observed at site 3 alone. Similarly, the most abundant species observed in UVS transects was *Chromis viridis* (Pomacentridae), which was almost exclusively found at site 7. The following species were abundant but restricted to one site only: *Cheilodipterus novemstriatus* (Site 3), *Nectamia annularis* (Site 3), *T*. *bilineata* (Site 3), *Pseudocheilinus hexataenia* (Site 6), *Eviota prasina* (Site 6), *C*. *viridis* (Site 7), *Pristotis cyanostigma* (Site 7). *Gnatholepis* sp. 2 (Site 8) and *Pseudocheilinus evanidus* (Site 8). The most abundant widely-spread species included *Pomacentrus albicaudatus*, *Trimma avidori*, *Asterropteryx semipunctata*, *Eviota zebrina*, *Eviota* sp. “Red Sea 3,” *Neopomacentrus cyanomos*, *Eviota distigma*, *Pomacentrus trilineatus*, and *Callogobius bifasciatus*.

**Fig 4:**
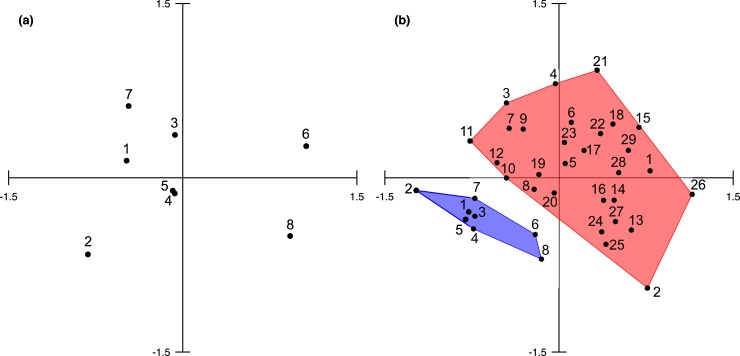
nMDS of Bray-Curtis dissimilarity distances. (a) Al Wajh sites based on both UVS and ichthyocide surveys. Stress: 0.1631. (b) Al Wajh sites (blue) and Coker et al. [29] sites (red) based only on ichthyocide surveys. Stress: 0.2064.

Between-site variability was visualized through an nMDS plot (Stress: 0.1631) (Fig 4A). The sampled sites clustered into two groups detected by SIMPROF analysis at a 95% confidence level. The first group was comprised of sites 1–5 and 7, whereas the second group was comprised of sites 6 and 8. These groups separate along the horizontal axis. There is an abundance gradient along the vertical axis, with more densely populated sites plotting at higher axis values. The second group sites were characterized by high abundance of acanthurids and gobies, and an absence of the otherwise abundant *P*. *trilineatus* and *N*. *cyanomos*. Richness and abundance at site 8 was greater than at site 6, but site 6 was more diverse (Table 2).

**Fig 5:**
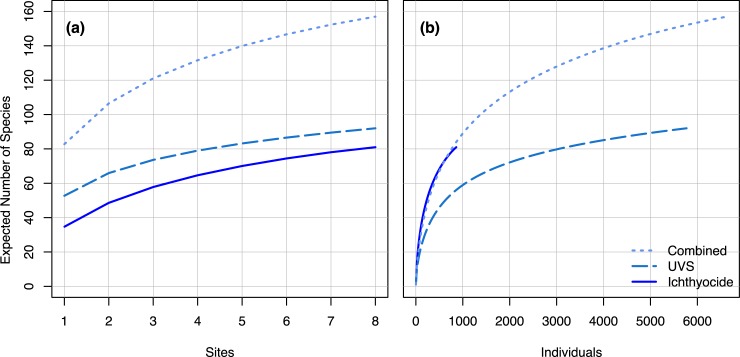
Rarefaction curves for UVS transects, ichthyocide stations, and combined results based on (a) sites and (b) individual fishes observed.

Rarefaction curves do not appear to approach a maximum, particularly in the site-based curves, suggesting that there were additional (undetected) species in Al Wajh Bank (Fig 5). The curves also showed that ichthyocide surveys accrue species more rapidly per each observed individual, and at a comparable rate to UVSs for each site. The Heltshe and Forrester jackknife estimator predicted the true species richness within Al Wajh Bank to be 214 using our methods, and that if we continued to sample, we would expect to find 127 species using UVS transects, and 120 species using ichthyocide stations. It should be noted that the variance of these jackknife estimates is quite large, 495.3 for UVS transects, 202.9 for ichthyocide stations, and 564.8 for the combined methods.

### Comparative community analysis

86% of the species observed in this UVS were also recorded by Roberts et al. [20]. 13 species were exclusive to Al Wajh Bank, and another 12 species occurred at a higher percentage of sites versus Roberts et al. [20] (Table 3). There was less overlap in fish assemblage between our ichthyocide survey and that of Coker et al. [29], with only 56% of species also observed outside Al Wajh and no overlap between clusters in the nMDS plot (Fig 4B). In both UVS and ichthyocide surveys, sites 6 and 8 shared more species with outside Al Wajh than with other sites within this study. Species distinguishing Al Wajh from other Red Sea reefs include planktivorous pomacentrids and caesionids including *Caesio striata*, *P*. *albicaudatus*, *P*. *trilineatus*, *P*. *cyanostigma*, *N*. *cyanomos*, *Neopomacentrus miryae*, and *Neopomacentrus xanthurus*, cryptobenthic species including *E*. *zebrina*, *Asterropteryx semipunctata*, and *T*. *avidori*, and the sand associated *Parupeneus forsskali*, *Taeniura lymma*, and *Torpedo sinuspersici* (Table 3, Table 4).

**Table 3:** SIMPER analysis of conspicuous species between our Al Wajh surveys and fish data obtained from Roberts et al. [20].

Species	Al Wajh Ave.	Roberts et al. [20] Ave.	Average/SD	Standard Deviation	Contribution %	Cumulative %
*Parupeneus forsskali*	87.5	0.0	2.55	0.003	0.65	0.65
*Cheilinus fasciatus*	75.0	0.0	1.69	0.003	0.56	1.20
*Pomacentrus trilineatus*	75.0	10.0	1.50	0.004	0.54	1.75
*Hemigymnus sexfasciatus*	75.0	10.0	1.50	0.004	0.54	2.29
*Neopomacentrus cyanomos*	75.0	10.0	1.50	0.004	0.54	2.83
*Pomacanthus asfur*	75.0	10.0	1.50	0.004	0.53	3.36
*Scolopsis ghanam*	62.5	0.0	1.28	0.004	0.48	3.84
*Cheilinus abudjubbe*	62.5	10.0	1.21	0.004	0.45	4.29
*Neopomacentrus miryae*	62.5	30.0	1.09	0.004	0.42	4.71
*Pomacanthus maculosus*	75.0	40.0	1.09	0.004	0.42	5.12
*Pomacentrus trichrourus*	62.5	40.0	1.03	0.004	0.40	5.52
*Chaetodon larvatus*	62.5	50.0	0.99	0.004	0.38	5.90
*Hemigymnus melapterus*	37.5	10.0	0.81	0.004	0.31	6.21
*Amblyglyphidodon flavilatus*	100.0	60.0	0.81	0.004	0.30	6.51
*Gerres* sp.	25.0	0.0	0.57	0.003	0.19	6.96
*Acanthopagrus bifasciatus*	25.0	0.0	0.57	0.003	0.19	7.15
*Caesio striata*	25.0	0.0	0.57	0.003	0.18	7.33
*Carangoides bajad*	25.0	0.0	0.57	0.003	0.17	7.50
*Epinephelus summana*	12.5	10.0	0.50	0.003	0.16	7.66
*Gymnothorax griseus*	12.5	0.0	0.37	0.002	0.10	7.76
*Scolopsis taeniatus*	12.5	0.0	0.37	0.002	0.10	7.86
*Fistularia commersonii*	12.5	0.0	0.37	0.002	0.10	7.96
*Dascyllus marginatus*	12.5	0.0	0.37	0.002	0.09	8.05
*Taeniura lymma*	12.5	0.0	0.38	0.003	0.09	8.14
*Torpedo sinuspersici*	12.5	0.0	0.38	0.003	0.09	8.23

Only fish species with greater percent occurrence in Al Wajh sites are shown.

**Table 4:** SIMPER analysis of cryptic species between our Al Wajh surveys and fish data obtained from Coker et al. [29].

	Al Wajh Ave.	Coker et al. [29] Ave.	Average/SD	Standard Deviation	Contribution %	Cumulative %
*Asterropteryx semipunctata*	87.5	24.1	1.31	0.016	2.06	2.06
*Eviota zebrina*	75.0	0.0	1.55	0.013	2.01	4.07
*Trimma avidori*	75.0	0.0	1.55	0.013	2.01	6.08
*Eviota* sp. “Red Sea 3”	75.0	10.3	1.39	0.014	1.91	7.99
*Pomacentrus trilineatus*	62.5	24.1	1.02	0.017	1.69	9.68
*Eviota guttata*	25.0	65.5	1.08	0.015	1.61	11.29
*Oxycheilinus* sp. 1	62.5	24.1	1.06	0.015	1.61	12.90
*Cheilodipterus pygmaios*	50.0	0.0	0.91	0.017	1.57	14.47
*Eviota distigma*	50.0	0.0	0.90	0.017	1.48	15.95
*Pomacentrus albicaudatus*	50.0	10.3	0.94	0.015	1.44	17.39
*Gobiodon reticulatus*	50.0	13.8	0.94	0.014	1.34	18.72
*Plesiops* sp. 1	0.0	44.8	0.83	0.016	1.33	20.05
*Gobiodon rivulatus*	25.0	44.8	0.88	0.015	1.31	21.36
*Callogobius bifasciatus*	50.0	3.4	0.95	0.014	1.29	22.66
*Cephalopholis hemistiktos*	50.0	3.4	0.96	0.013	1.29	23.94
*Apogon coccineus*	37.5	31.0	0.85	0.015	1.26	25.20
*Fowleria variegata*	37.5	0.0	0.72	0.017	1.25	26.45
*Amblyglyphidodon flavilatus*	37.5	3.4	0.72	0.017	1.25	27.70
*Larabicus quadrilineatus*	37.5	17.2	0.80	0.015	1.19	28.90
*Enneapterygius* sp. 1	37.5	3.4	0.75	0.015	1.09	29.99
*Neopomacentrus xanthurus*	37.5	0.0	0.74	0.014	1.03	31.02
*Cabillus*. sp. 1	37.5	0.0	0.75	0.014	1.03	32.05
*Istigobius* sp. 2	37.5	3.4	0.76	0.013	1.01	33.06
*Eviota* cf. *pardalota*	37.5	0.0	0.75	0.013	0.99	34.05
*Gobiidae* sp. 10	0.0	34.5	0.68	0.014	0.94	34.99
*Plectroglyphidodon lacrymatus*	0.0	34.5	0.68	0.014	0.93	35.92
*Ecsenius frontalis*	25.0	20.7	0.71	0.013	0.91	36.83
*Blenniidae*. sp. 2	0.0	34.5	0.69	0.013	0.90	37.73
*Antennatus*.*coccineus*	25.0	20.7	0.70	0.013	0.89	38.62
*Pseudochromis flavivertex*	25.0	3.4	0.55	0.016	0.86	39.49
*Tripterygiidae* sp. 1	0.0	31.0	0.62	0.014	0.84	40.33
*Thysanophrys cf*. *chiltonae*	25.0	3.4	0.58	0.014	0.82	41.15
*Pseudocheilinus hexataenia*	25.0	17.2	0.69	0.012	0.81	41.96
*Priolepis cincta*	25.0	13.8	0.66	0.012	0.79	42.75
*Neamia octospinus*	12.5	27.6	0.68	0.011	0.78	43.52
*Pseudochromis olivaceus*	12.5	24.1	0.64	0.012	0.77	44.30
*Dascyllus marginatus*	25.0	3.4	0.58	0.013	0.77	45.06
*Fusigobius* sp. 1	12.5	24.1	0.64	0.012	0.76	45.82
*Ostorhinchus nigrofasciatus*	0.0	27.6	0.56	0.013	0.74	46.56
*Pristotis cyanostigma*	25.0	0.0	0.55	0.013	0.73	47.29
*Eviota* sp. “Red Sea 2”	0.0	27.6	0.58	0.013	0.72	48.01
*Antennatus*.*nummifer*	25.0	0.0	0.56	0.012	0.69	48.71
*Pomacentridae* sp. 1	0.0	24.1	0.52	0.013	0.69	49.39
*Gnatholepis* sp. 1	12.5	17.2	0.55	0.012	0.64	50.04

Only showing species contributing ≥50% of the observed differences. Species with greater percent occurrence outside Al Wajh sites greyed out.

### Benthic cover

Reef habitats across Al Wajh Bank were dominated by two major categories–hard coral and sand (Table 5). 95% of the hard corals belonged to one of four families–Acroporidae, Poritidae, Pocilloporidae, and Merulinidae. Overall, Poritidae were the most abundant, being particularly dominant at sites 1, 3, and 5, whereas Acroporidae were dominant at site 7 and Pocilloporidae were dominant at site 6. The remaining 5% of hard corals included members of the Agariciidae, Fungiidae, the Euphylliid genus, *Galaxea*, and the non-scleractinian fire corals (*Millepora*). Soft corals comprised 5% of the benthic habitat, 72% of which were within Xeniidae. About 3% of the benthos was occupied by sponges. Rubble & pavement was most abundant at site 8, whereas turf algae was most abundant at site 6. Dead coral consistently comprised about 3% of the benthos. We expected to observe a higher frequency of dead coral following a major bleaching event in 2015 that had occurred only a few months before sampling and impacted other Red Sea reefs [39].

**Table 5:** Composition of benthic habitat in Al Wajh Bank across each site.

	1	2	3	4	5	6	7	8	Total
Hard coral	28.7 ±2.9	50.7 ±6.4	35.3 ±7.9	49.3 ±6.4	57.3 ±1.3	22.0 ±2.3	39.3 ±1.3	33.3 ±9.0	39.5 ±4.3
*Porites*	16.7 ±2.9	7.3 ±0.7	20.0 ±3.1	14.7 ±4.8	32.7 ±2.4	0.7 ±0.7	1.3 ±1.3	13.3 ±5.8	13.3 ±3.7
*Acropora*	2.0 ±2.0	8.7 ±2.4	2.7 ±0.7	11.3 ±2.4	2.0 ±2.0	2.7 ±2.7	29.3 ±1.8	7.3 ±0.7	8.3 ±3.3
Merulinidae	4.7 ±0.7	10.0 ±1.2	8.7 ±3.5	10.7 ±1.8	4.7 ±1.8	0.7 ±0.7	4.0 ±2.0	6.0 ±1.2	6.2 ±1.2
*Stylophora*	2.7 ±2.7	7.3 ±1.3	2.7 ±1.8	4.7 ±2.9	5.3 ±2.7	15.3 ±0.7	2.7 ±1.3	4.0 ±2.0	5.6 ±1.5
*Montipora*	0.7 ±0.7	10.0 ±4.2	0.7 ±0.7	7.3 ±0.7	7.3 ±2.9	0.0	1.3 ±0.7	0.7 ±0.7	3.5 ±1.4
Agariciidae	0.7 ±0.7	6.0 ±3.1	0.0	0.0	0.0	0.7 ±0.7	0.0	0.0	0.9 ±0.7
*Galaxea*	0.7 ±0.7	0.7 ±0.7	0.0	0.7 ±0.7	3.3 ±2.4	0.0	0.0	0.0	0.7 ±0.4
*Millepora*	0.0	0.7 ±0.7	0.7 ±0.7	0.0	0.0	1.3 ±0.7	0.7 ±0.7	0.7 ±0.7	0.5 ±0.2
*Pocillopora*	0.0	0.0	0.0	0.0	0.0	0.7 ±0.7	0.0	1.3 ±1.3	0.3 ±0.2
Fungiidae	0.7 ±0.7	0.0	0.0	0.0	0.7 ±0.7	0.0	0.0	0.0	0.2 ±0.1
*Seriatopora*	0.0	0.0	0.0	0.0	1.3 ±1.3	0.0	0.0	0.0	0.2 ±0.2
Sand	36.0 ±3.1	32.0 ±10.1	47.3 ±5.5	19.3 ±4.1	8.0 ±1.2	43.3 ±1.8	52.0 ±3.5	16.7 ±13.8	31.8 ±5.6
Pavement/rubble	14.7 ±2.9	6.0 ±2.3	9.3 ±1.3	20.7 ±3.5	6.7 ±1.3	3.3 ±0.7	0.7 ±0.7	27.3 ±7.0	11.1 ±3.2
Turf algae	5.3 ±2.4	0.0	0.0	1.3 ±1.3	2.0 ±1.2	27.3 ±1.3	2.0 ±1.2	7.3 ±2.4	5.7 ±3.2
Soft coral	0.0	0.0	5.3 ±1.3	2.0 ±0.0	20.0 ±6.1	0.0	4.0 ±2.0	8.7 ±4.8	5.0 ±2.4
Sponge	9.3 ±1.3	6.0 ±2.3	1.3 ±0.7	3.3 ±0.7	3.3 ±2.4	1.3 ±0.7	0	2.0 ±1.2	3.3 ±1.1
Dead coral	2.0 ±1.2	3.3 ±1.3	0.7 ±0.7	4.0 ±1.2	2.7 ±1.8	2.7 ±0.7	2.0 ±1.2	4.7 ±1.8	2.8 ±0.4
Other	4.0 ±2.0	2.0 ±1.2	0.7 ±0.7	0.0	0.0	0.0	0.0	0.0	0.8 ±0.5

Average percent cover calculated from each site (numbered columns) and grand mean from all sites (“Total”) with standard error. Categories are listed in order of descending percent cover. Coral genera belong to the families Poritidae (*Porites*), Acroporidae (*Acropora*, *Montipora*), Pocilloporidae (*Pocillopora*, *Seriatopora*, *Stylophora*), Euphylliidae (*Galaxea*), and Milleporidae (*Millepora*).

## Discussion

Al Wajh Bank is isolated and environmentally distinct from other Red Sea reefs, however its remote location has limited field studies within the area. Moreover, potential changes in the reef’s undocumented communities caused by development necessitates the need for baseline ecological data. Most of the biodiversity surveys in the northern Red Sea have been conducted in the Gulf of Aqaba [40–42], 350km northeast of Al Wajh. Few more proximal reefs have been sampled [20] but none from within the bank itself. The present study fills this knowledge gap for both conspicuous and cryptobenthic reef fishes. Use of combined UVS and ichthyocide collections aided our sampling effort by augmenting the rate of species detection. Even within an environment that experiences greater fluctuations in temperature and higher turbidity levels when compared to outside the bank, coral cover is relatively high (~40%) and associated fish communities remain diverse and abundant. These surveys help provide a baseline for this area and an idea of how important and unique some of these unexplored regions are for fish communities.

Roberts et al. [20] reported that fish communities along the Saudi Arabian coast of the Red Sea were homogenous. We were unable to test if this pattern holds true for conspicuous fishes in Al Wajh, however, SIMPER analysis detects several conspicuous species that are were found exclusively or more frequently in Al Wajh (Table 3). nMDS comparison of our ichthyocide-collected samples with those from Coker et al. [29] (Fig 4B) does suggest that cryptobenthic communities are distinct from other Red Sea reefs. Cryptobenthic species may be more sensitive to the scale of environmental variability that distinguishes Al Wajh Bank, but uniqueness in both fish categories is still a possibility. Species composition at sites 6 and 8, located along the periphery of Al Wajh lagoon, does appear to be an intermediate between communities inside and outside the lagoon for both cryptobenthic and conspicuous fishes. If fish communities in Al Wajh are unique, then there may be other ecologically distinct regions in the Red Sea that could be detected using similar methods of sampling. Areas adjacent to the Farasan Islands (southern Red Sea) have the potential to produce similar environmental conditions. This conclusion must, however, be taken with caution due to the low number of sites and differences in sampling methodology between the studies being compared.

**Fig 6:**
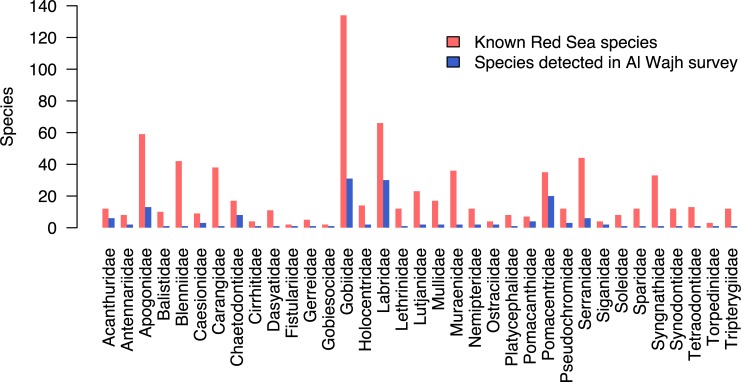
Comparison of species diversity in each fish family for both survey methods against known species in the Red Sea based on Golani and Fricke [43].

The most complete checklist of fishes in the Red Sea lists 1207 species [43], but this number is constantly being updated [44]. Our survey efforts documented 153 species and OTUs from the Al Wajh Bank region, 71% of the total species richness estimated by the jackknife estimator. Compared to all the known Red Sea fishes, the species in this study account for a small fraction, even when only considering reef-associated species, but there is high variation in representation from each family (Fig 6). Al Wajh had highest representation (45–57%) from the Pomacentridae, Pomacanthidae, Acanthuridae, Chaetodontidae, Labridae, Siganidae, Ostraciidae, Gobiesocidae, and Fistulariidae. We collected 16 OTUs, 10% of the total number of species collected, most of them (*N* = 12) from the family Gobiidae. It is possible these names represent described species that our team was unable to identify, but there exists potential for some to be undescribed. The OTUs *E*. sp. *“Red Sea 1”* and *E*. sp. *“Red Sea 3”* were first documented in 2013 [45], but require formal species descriptions.

Based on the rarefaction curves (Fig 5) and species richness estimates, we also hypothesize that additional sampling will provide greater accuracy in representing the fish community, but we cannot predict if and how this would change the variation between families. Note that our survey occurred in the winter. It is possible that the fish community changes throughout the year since the water temperature varies seasonally, and can affect metabolic rate, oxygen solubility, and availability of other resources. Thus, we recommend that future surveys sample throughout the year. While this study provides the first biodiversity assessment at Al Wajh Bank, we do not examine invertebrate diversity. Invertebrate surveys should be prioritized, as uniqueness in both fish and invertebrate communities could arise if subject to the same environmental conditions.

The extreme physical properties of Al Wajh Bank provide a unique opportunity to study how corals live in stressful conditions [46]. Under severe and/or prolonged stress, corals bleach, releasing their endosymbiotic algae [47], but some species have a higher tolerance than others [46, 48, 49]. Additionally, entire coral reefs are found throughout a range of potentially stressful conditions. In the Red Sea, corals have built extensive reefs in temperatures thought to be near their physiological limits. These Red Sea corals are frequently studied due to their apparent tolerance to high temperature and salinity [50–52]. The corals in Al Wajh are subject to even more of these extremes and may provide important case studies related to coral bleaching and stress tolerance in the future.

In 2017, Saudi Arabia announced the launch of The Red Sea tourism project as a part of Saudi Vision 2030 [25, 26]. The project aims to create a resort with hotels and luxury residential units planned to be built across 50 islands within Al Wajh Bank. Construction began in late 2018 and the resort is expected to be partially operational by 2022. Ecotourism has the potential to provide alternative revenue in Saudi Arabia’s fossil fuel-driven economy, and increase awareness of marine life in regional culture, but habitat loss and alterations from construction and tourism can be expected [53, 54]. Corals support fishes through the provision of food and habitat resources [55, 56], therefore a reduction in abundance and diversity of coral habitats and reef structure is expected to be detrimental to associated fish communities [57]. The most immediate responses will likely be seen in the cryptobenthic and other highly coral-associated species [58], but any disturbance has the potential to cascade through the trophic web [59]. While construction is in progress, access to Al Wajh Bank will be severely limited, thus, our research team has no immediate plans to return to Al Wajh Bank, but we strongly encourage follow-up surveys at the next available opportunity to continue documenting changes in regional biodiversity.

Fishing activity at Al Wajh Bank is also present, but its broader impact is largely unknown. Fishermen likely arrive from ports in the nearby towns of Al Wajh and Yanbu. Low accessibility to Al Wajh’s remote reefs may likely result in low fishing pressure, but fishing may also be encouraged by infrequent coastguard patrols [24]. Along the Saudi Arabian coast of the Red Sea, including at Al Wajh Bank, there is a conspicuous lack of large predators. This is thought to be attributed to unregulated fishing efforts, but historical data are unavailable to confirm this hypothesis [60, 61]. Fishing activity is likely to decrease once development of the Red Sea tourism project begins.

Al Wajh Bank is unique in its physical properties and supports a fish community distinct from other Red Sea reefs. We recognize the region as a new type of reef habitat in the Red Sea, characterized by its unique bathymetry and water conditions. The ecological role of Al Wajh in the greater Red Sea is largely unknown, however habitat diversity positively correlates to species diversity [62], and both may play a role in the long-term stability of Red Sea reefs. Diversity of species provides redundancies in ecological roles. Diversity in habitat could make additional resources available to some species, and different habitats may support different stages of development, such as mangrove forests acting as nursery habitats for reef species [63]. Al Wajh Bank could be serving a similar nursery role.

## Conclusion

The body of research on coral reefs in the Red Sea is small in comparison to other reef systems, and few studies consider local variation along the Red Sea’s 12° of latitude, but existing work depicts the region as high in diversity and endemism. The Kingdom of Saudi Arabia is undergoing a period of rapid social and economic change, and marine life in the Red Sea is becoming increasingly intertwined in these changes. The lack of historical data, however, makes it difficult to document anthropogenic changes and apply appropriate management strategies. Al Wajh Bank is expected to be transformed into a tourist hotspot in the coming years, and the health and vibrancy of its ecosystem is vital to research on extreme conditions found in the Red Sea and the long-term economic success of the region. Here, we provide baseline data on the fish community and other characteristics of Al Wajh Bank to inform future research and decisions that may affect the reef. The authors recommend that future surveys in this area continue to use combined UVS-ichthyocide surveys to capture taxonomic breadth and for comparability with this study. It is also advised that additional surveys be conducted during summer months to detect potential seasonal differences in the reef community.

## Supporting information

S1 Table Genetic verification of cryptobenthic samples. (XLSX)

S2 Table Fish counts for all species, methods, sites, and transects. (XLSX)

S3 Table Comparing OTUs used across current study, Coker et al. 2018, and Isari et al. 2017. Matches were determined using COI sequences with a > 96% match.
(XLSX)
